# OP33 The Voice Of Patients And Caregivers: Social Impact Of Rare Diseases In Health Technology Assessment In Ecuador

**DOI:** 10.1017/S0266462325100913

**Published:** 2025-12-29

**Authors:** Veronica Simbaña

## Abstract

**Introduction:**

Rare diseases, including Gaucher disease and spinal muscular atrophy, impose significant challenges in access to treatments and equitable healthcare policies. In Ecuador, patients and caregivers lack representation in health technology assessment (HTA) processes. This study aimed to amplify their voices, exploring their experiences to develop inclusive methodologies that promote equity and transparency in healthcare decision-making.

**Methods:**

This study employed a mixed-methods approach combining qualitative and quantitative data comprising perspectives of patients with Gaucher disease and spinal muscular atrophy (as well as the perspectives of their caregivers), in-depth interviews, surveys, and observation. Collaboration occurred with patient organizations. Qualitative analysis focused on thematic coding of experiences, while quantitative surveys assessed access to treatment and socioeconomic burdens. Data triangulation ensured robust findings.

**Results:**

Preliminary results indicated significant challenges in equitable access to treatments for rare diseases in Ecuador. Patients and caregivers reported high emotional and financial burdens, with limited systemic support. Early findings suggested disparities in treatment availability and awareness between urban and rural settings. Engagement workshops demonstrated a strong willingness among stakeholders to participate in developing inclusive HTA processes.

**Conclusions:**

Preliminary findings highlighted the urgent need for inclusive HTA processes in Ecuador. Amplifying patient and caregiver voices can improve equity in rare disease management and inform policy changes. Future research will focus on refining methodologies for participatory HTA and addressing systemic gaps in access to treatment, with emphasis on rural and vulnerable populations.

